# Mixed *Leucaena* and molasses can increase the nutritional quality and rumen degradation of corn stover silage

**DOI:** 10.5455/javar.2023.j660

**Published:** 2023-03-31

**Authors:** Yusuf Akhyar Sutaryono, Ryan Aryadin Putra, Mardiansyah Mardiansyah, Enny Yuliani, Harjono Harjono, Mastur Mastur, Sukarne Sukarne, Luh Sri Enawati, Dahlanuddin Dahlanuddin

**Affiliations:** 1 Faculty of Animal Science, University of Mataram, Mataram, Indonesia; 2 Vocational School, University of Mataram, Mataram, Indonesia; 3 Faculty of Animal Husbandry, Marine and Fisheries, University of Nusa Cendana, Kupang, Indonesia

**Keywords:** Corn stover, silage, Leucaena, watersoluble, carbohydrate

## Abstract

**Objective:**

The study was conducted to determine the effect of *Leucaena* at different proportions and doses of molasses on the nutritional quality, silage fermentation characteristic, and *in vitro* digestibility of corn stover silage.

**Materials and Methods:**

The study was designed in a completely randomized factorial design 3*3 pattern. The first factor was the proportion addition of *Leucaena*, i.e., L0 (0%), L15 (15%), L30 (30%), and L45 (45%) of inclusion of *Leucaena* on the dry matter (DM) basis of corn stover. The second factor was the dose of inclusion of molasses, i.e., M2 (2%), M4 (4%), and M6 (6%) on the fed basis of silage. Each treatment had five replications. The variables observed included chemical composition [DM, organic matter (OM), crude protein (CP), crude fiber (CF), hemicellulose, acid detergent fiber, and neutral detergent fiber], silage fermentation characteristics (pH and NH_3_-N), DM digestibility (DMD), and OM digestibility (OMD) under *in vitro* conditions.

**Results:**

The result shows that the inclusion of *Leucaena* in the proportion of 30%–45% is very effective in increasing and improving the chemical composition of corn stover silage, significantly suppresses the content of CF, and increases the CP content of the silage. Likewise, the inclusion of molasses at a dose of 4% also positively contributed to the quality of the resulting silage, especially its effect in suppressing the buffer capacity of proteins resulting in low pH values and NH_3_-N concentrations in silage.

**Conclusions:**

It was concluded that the inclusion of *Leucaena* in 30%–45% and the inclusion of molasses at a dose of 4% is very effective in increasing and improving the chemical composition, silage fermentability characteristics, and rumen degradation of corn stover silage.

## Introduction

Feed availability for cattle in West Nusa Tenggara Province fluctuates between the rainy and dry seasons. Due to the erratic seasonal rainfall in this area, it causes forage throughout the year to fluctuate in quantity and quality [1], and those fluctuations of the growth and body weight of Bali cattle raised by farmers in this province. With high feed availability in the rainy season, the body weight growth is very high, but during the dry season, the body weight of cattle will decrease rapidly due to less quantity and quality of feed availability [2].

The high fluctuation of feed availability in this region needs to be addressed with the use of feed that is available in large numbers, easy to be accessed, and cheap. The most widely known in this region is corn. The availability of corn stover in this region is extensive due to the large amount of land planted with corn. Until recently, most of this corn stover was left wasted, returned to the soil, and burned [3]. Burning this biomass destroys organic matter (OM) potential for cattle feed [4], and causes massive environmental pollution due to the high carbon released into the atmosphere [5].

The use of corn stover as feed has drawbacks; approximately 50%–70% of corn stover is composed of cellulose, hemicellulose, and lignin, affecting the utilization efficiency [6]. Because of its low protein content, so it needs to be mixed with other high protein feeds, one of which is widely adopted and used as cattle feed in this area is *Leucaena leucocephala cv. Tarramba* [7,8]. Adding protein is expected to respond positively [9]. However, adding high-protein materials in the silage has its problems. The problem is low dry matter (DM) content, water-soluble carbohydrate (WSC) concentration, and high buffering capacity, mainly when harvesting [10]. Hence, it is necessary to consider adding WSC to overcome them. The most widely available WSC at low prices is molasses. The addition of molasses can provide a fast carbohydrate substrate for lactic acid bacteria (LAB) in producing lactic acid [11], and silage fermentation efficiency can be achieved [12], and finally can improve livestock performance [13,14]. The study aimed to test the effect of several additional levels of *Leucaena* and the doses of molasses in increasing and improving the chemical composition, silage fermentability characteristics, and digestibility of corn stover silage.

## Materials and Methods

### Silage preparation process

The material used in this experiment is corn stover, *Leucaena*, and molasses. Corn stover was collected randomly from corn stover fields in the Central Lombok district, West Nusa Tenggara Province, Indonesia, while molasses was obtained from a molasses trader in Mataram city. Corn stover and *Leucaena* leaves were then chopped to 3–6 cm in size. Before chopping the corn stover, *Leucaena* leaves were let dry under the shade for 6 h to achieve a water content of approximately 65%. The experiment was conducted on a laboratory scale. Silage was prepared from mixed corn stover and *Leucaena* in a 5 kg mixture, with a *Leucaena* proportion of 0%, 15%, 30%, and 45% of the total mix. Molasses were applied in 2%, 4%, and 6% doses into the corn stover and *Leucaena*. All materials were mixed well, placed into a plastic container, pressed and vacuumed to reduce oxygen in the silo, and then sealed. Finally, all silos (plastic containers) were placed in a sterile room and left for fermentation for 21 days before being harvested.

### Sample analysis procedure

Before the ensiling process, the procedure of [15] was applied to calculate the DM, OM, CP, and CF content. The method described by Van Soest et al. [16] has been applied to analyze the content of hemicellulose, neutral detergent fiber (NDF), and acid detergent fiber (ADF). Fermentation characteristics were analyzed based on pH and NH_3_-N. Analysis of pH silage followed the procedure [17] using a pH meter (Metrohm 691 with pH electrode). Analysis of NH_3_-N concentration follows the procedure of [18] using a spectrophotometer with a reading wavelength of 640 nm. *In vitro* digestibility was analyzed using the methods developed in [19]. *In vitro* tubes were filled with samples consisting of rumen fluid and artificial saliva solution (McDougal solution) with 1:4 ratios. Carbon dioxide (CO_2_) gas was provided simultaneously to enable the anaerobic condition in tubes that will be incubated. Incubation was conducted in the water bath at 39°C–40°C for 48 h.

### Experimental design

The study was designed in a completely randomized design with 3*3 factorial pattern. The first factor was the proportion addition of *Leucaena*, as follows: L0 (0%), L15 (15%), L30 (30%), and L45 (45%) of inclusion of *Leucaena* on the DM basis of corn stover. The second factor was the dose of inclusion of molasses, as follows: M2 (2%), M4 (4%), and M6 (6%) on the fed basis of silage. Each treatment had five replications. Hence, there were 60 experimental units.

### Data analysis

The variables observed included the chemical composition of silage (DM, OM, CP, CF, hemicellulose, ADF, and NDF), silage fermentation characteristics (pH and NH_3_-N), dry matter digestibility (DMD), and organic matter digestibility (OMD) under *In vitro* conditions. All data obtained were then processed with Statistical Product and Service Solutions software version 20 based on the design used. If there are differences between treatments, Duncan's New Multiple Range Test was applied.

## Results

### Effect on the chemical composition of silage

The results showed that the increase of *Leucaena* proportion substantially affects the value of DM, CP, CF, hemicellulose, ADF, and NDF of corn stover silage (*p* < 0.05; Table 2). Specifically, the increase of *Leucaena* increased the CP content and decreased CF and its fractions. The CP content in L0, L15, L30 and L45 were 6.11%, 7.98%, 8.85% and 11.09% respectively (*p* < 0.05).

A similar result is shown by adding a dose of molasses, where increasing the dose significantly affected corn stover silage nutrient, except for hemicellulose content. The most potent effect of molasses was indicated by the NDF value increase of 64.54%, 62.92%, and 60.76% for M2, M4, and M6, respectively (*p* < 0.05; Table 2). A significant effect of interaction between *Leucaena* and molasses was also shown by DM, OM, CP, CF, hemicellulose, and NDF (*p* < 0.05).

### Effect on silage fermentability quality

The experiment showed no effect of the interaction of *Leucaena* and molasses on the fermentation quality of silage. Although the addition of *Leucaena* and molasses significantly affected the pH value and NH_3_-N concentration of silage (*p* < 0.05; Table 2). Based on the partial effect, the pH value increased substantially in line with the increase of *Leucaena* proportion in the silage (3.58–4.07 on average; *p* < 0.05), but there was a significant decrease when molasses was added (3.87–3.70 on average; *p* < 0.05) with the lowest value is in L0 treatment.

The increase of *Leucaena* proportion increased the NH_3_-N concentration of silage (*p* < 0.05), the value of NH_3_-N concentration caused by the increase of *Leucaena* proportion was 5.2 (L0), 6.24 (L15), 6.90 (L30), and 8.27 mg/100 ml (L45) (Table 2). On the other hand, the NH_3_-N concentration decreased with the increase of molasses dose (*p* < 0.05) with pH values of 7.87, 6.61, and 5.76 mg/100 ml for M2, M4, and M6 respectively.

### Effect on DMD and OMD

DMD and OMD of the silage increased concomitantly with the increased proportion of *Leucaena* and molasses dose (*p* < 0.05). Moreover, a significant interaction between *Leucaena* and molasses (*p* < 0.05) affected the DMD and OMD of silage. DMD and OMD of silage increased significantly with the *Leucaena* proportion of 45% of total silage (49.10% and 50.87%, respectively). The DMD and OMD values with the additional dose of molasses at 2% were 42.63% and 44.37%, while 4% were 44.06% and 45.40% (*p* < 0.05). 

## Discussion

### Chemical composition of silage

The result showed the significant effect of *Leucaena* and molasses addition on the silage quality (*p* < 0.05). The increase in DM content was the direct effect of the *Leucaena* addition on the silage. The increasing DM content obtained in this study is clearly due to increased CP content silage (Table 2). In this regard, mixing several materials with high DM content in silage or feed-making could increase the DM content compared to the DM content used as single feed material. Another researcher also reported a similar result in increased DM content in silage when the proportion of legume (Cowpea) is added [20]. The increase in DM silage content due to increased molasses dosage is thought to be due to the high contribution of single-cell protein from LAB (indicated by increased silage CP, Table 2), which may have an overall impact on DM silage content when chemical composition analysis is carried out. However, in our study, no investigation was carried out on the population and epiphytic diversity of LAB that grew during the ensiling process. The results study by Rambau et al. [21] also showed an increase in DM silage due to the combined effect of bio slurry-digester with molasses. Silage with a high DM content shows that the nutrient contained also increases; for example, this study shows an increase in CP and energy silage. Silage with high protein and energy content is identified as a quality feed that can optimize cattle growth. By increasing silage quality, the amount of silage consumed by cattle will increase as the quality of silage increases and vice versa; when there is a decrease in quality, the amount consumed will also decrease. 

Table 2 shows that the OM content decreased slightly in line with an increased dose of molasses (*p* < 0.05), contrary to the effect of molasses dose that increases the DM content of the silage. The decrease of OM is affected by the increase of molasses dose possibility caused by using several nutrients by LAB into a soluble product during the ensiling process. The rate of the LAB population increase during the ensilage process might be very high; hence there was a need for high energy that caused the high use of OM. The increased number of LAB causes their nutritional needs to increase [22]. Microorganisms need essential nutrients, especially energy sources, to support cell multiplication [23]. In the anaerobic fermentation process, fermented sugar is used in high amounts during the intensive fermentation phase during the aerobic respiration period, but when the fermentation process enters the stable phase, the demand for the substrate is reduced [24]. The DM and OM content of corn stover silage showed significant differences due to the interaction effect between *Leucaena* and molasses. The best interaction was shown at the treatment of 45% *Leucaena* and 6% molasses with a DM value of 95.97% (*p* < 0.05). 

In all treatments, increasing CP content was observed (*p* < 0.05; Table 2). The increase in protein content with increasing *Leucaena* content in silage can be explained by the fact that the increase in CP content in silage is a direct effect of increasing the proportion of *Leucaena*, which is known to have a high CP content of 23.47% compared to the CP content of corn stover, which is 5.48% (Table 1). Thus, in this case, mixed feed materials have an associative effect, where increasing the proportion of *Leucaena* leads to a linear increase in CP content. This result was confirmed by [25], who reported that adding *Leucaena* to 37% of the prepared cactus silage produced the highest nitrogen.

**Table 1.** Chemical composition of *L. leucocephala cv. Tarramba* and corn stover.

Chemical Composition	Corn stover	*Leucaena*
DM, %	89.92	87.53
OM, %	94.12	91.53
CP, %	5.48	23.47
CF, %	30.56	20.16

**Table 2.** Nutrient composition, characteristics fermentative and *in vitro* digestibility of mixture silage corn stover with different inclusion of *Leucaena* and doses of molasses.

Variable	L0			L15			L30			L45			SEM	*p-*value		
	M2	M4	M6	M2	M4	M6	M2	M4	M6	M2	M4	M6		L	M	L × M
Nutrient composition (%)																
DM	92.50 ± 0.24^a^	92.93 ± 0.77^a^	94.13 ± 0.51^c^	93.94 ± 0.48^bc^	93.17 ± 0.56^ab^	95.74 ± 0.49^d^	95.96 ± 0.45^d^	95.60 ± 0.61^d^	95.11 ± 0.62^d^	95.97 ± 0.65^d^	95.36 ± 0.54^d^	95.96 ± 0.17^d^	0.309	<0.001	0.001	<0.001
OM	91.68 ± 0.65^c^	91.53 ± 0.56^bc^	90.19 ± 1.11^a^	91.48 ± 0.22^bc^	90.61 ± 0.23^ab^	90.88 ± 0.50^abc^	91.72 ± 0.25^c^	91.65 ± 0.40^c^	91.23 ± 0.15^bc^	91.66 ± 0.30^c^	90.96 ± 0.23^abc^	91.72 ± 0.51^c^	0.288	0.093	0.014	0.032
CP	5.21 ± 0.35^a^	6.52 ± 0.17^b^	6.60 ± 0.13^b^	6.94 ± 0.12^c^	8.20 ± 0.34^d^	8.78 ± 0.19^f^	8.31 ± 0.13^de^	8.60 ± 0.16^ef^	9.64 ± 0.29^g^	10.42 ± 0.17^h^	10.95 ± 0.18^i^	11.89 ± 0.16^j^	0.114	<0.001	<0.001	<0.001
CF	31.49 ± 0.65^g^	30.59 ± 0.17^f^	29.41 ± 0.50^e^	29.75 ± 0.34^e^	28.00 ± 0.35^d^	27.79 ± 0.21^cd^	27.64 ± 0.34^cd^	26.09 ± 0.77^b^	25.55 ± 0.32^b^	27.17 ± 0.47^c^	25.67 ± 0.32^b^	23.33 ± 0.32^a^	0.251	<0.001	<0.001	0.002
Hemmicelulose	24.09 ± 0.36^g^	22.30 ± 0.91^def^	22.20 ± 1.37^cde^	22.59 ± 1.12^defg^	23.21 ± 0.40^efg^	23.94 ± 0.49^fg^	21.17 ± 0.53^cd^	22.91 ± 0.52^efg^	21.60 ± 1.69^cde^	18.52 ± 0.61^a^	20.58 ± 0.73^bc^	19.46 ± 0.84^ab^	0.515	<0.001	0.204	0.008
ADF	44.37 ± 0.46	43.01 ± 0.78	41.10 ± 0.93	43.17 ± 0.54	41.23 ± 0.36	39.59 ± 0.16	42.47 ± 0.64	39.84 ± 0.57	38.65 ± 0.88	41.73 ± 0.14	38.56 ± 0.25	36.46 ± 0.14	0.325	<0.001	<0.001	0.052
NDF	68.47 ± 0.42^h^	65.32 ± 0.15^fg^	63.30 ± 0.64^d^	65.76 ± 0.81^g^	64.44 ± 0.46^ef^	63.53 ± 0.48^de^	63.63 ± 0.56^de^	62.76 ± 0.55^d^	60.25 ± 0.81^c^	60.25 ± 0.61^c^	59.15 ± 0.59^b^	55.92 ± 0.88^a^	0.341	<0.001	<0.001	0.001
Silage fermentation characteristic																
pH value	4.11 ± 0.12	4.12 ± 0.14	3.98 ± 0.17	4.02 ± 0.19	3.90 ± 0.18	3.77 ± 0.17	3.73 ± 0.21	3.70 ± 0.10	3.51 ± 0.16	3.60 ± 0.19	3.59 ± 0.16	3.55 ± 0.13	0.075	<0.001	0.008	0.835
NH_3_-N(mg N/ml)	6.45 ± 1.66	4.89 ± 1.00	5.22 ± 1.36	6.95 ± 0.86	6.76 ± 1.06	4.99 ± 0.79	8.49 ± 1.20	6.93 ± 1.03	5.28 ± 1.47	9.45 ± 1.66	7.84 ± 1.53	7.52 ± 1.22	0.570	<0.001	<0.001	0.365
*In vitro* rumen digestibility (%)																
DMD	38.10 ± 0.45^a^	38.65 ± 0.44^ab^	39.56 ± 0.28^b^	41.82 ± 0.62^c^	43.90 ± 0.52^d^	46.15 ± 0.38^e^	43.67 ± 0.72^d^	45.82 ± 0.12^e^	47.34 ± 0.56^f^	46.95 ± 0.75^ef^	49.11 ± 0.88^g^	51.24 ± 0.54^h^	0.324	<0.001	<0.001	0.001
OMD	40.12 ± 0.64^a^	39.03 ± 0.60^a^	41.56 ± 0.28^b^	42.31 ± 0.51^b^	45.01 ± 0.78^c^	46.93 ± 0.76^de^	45.24 ± 0.40^c^	46.78 ± 0.59^d^	48.12 ± 0.98^ef^	48.54 ± 1.06^f^	50.77 ± 1.09^g^	53.30 ± 0.37^h^	0.431	<0.001	<0.001	0.001

The increasing protein content of silage also occurs caused by the increased number of additive molasses applied. The higher the molasses, the higher the CP content of the silage. These results were associated with LAB growth as a direct amount of molasses source for WSC in the silage. An increased dose of molasses means more energy is available for LAB to grow and proliferate. The high proliferation rate of LAB would contribute to the increase in the CP content of silage through the contribution of their single-cell protein. The dead bacteria will also be considered CP when analyzing the CP content of the silage. The current results are in contrast to those found by Rambau et al. [21], who reported a substantial numerical reduction in CP silage in their study by adding fermentable carbohydrate additives. The highest CP content at 11.89% found in this experiment is enough to fulfill cattle needs for maintaining their life. Furthermore, Putra et al. [26] stated that to meet the CP requirement of cattle, it requires a minimum of 12% CP content in its ration.

The low CF content of silage with *Leucaena* proportion of 45% is probably by the increase of *Leucaena* proportion in the corn stover silage. *Leucaena* has lower CF content than corn stover (20.16 *vs.* 30.56, Table 1); hence, the higher the *Leucaena* added, the lower the CF. The CF content of corn stover-only silage was 30.50%, while the silage with the proportion of *Leucaena* 15%, 30%, and 45% was 28.52%, 26,43%, and 25.39%, respectively (*p* < 0.05). This result agreed with Bureenok et al. [27], who reported a decrease in the content of the fiber fraction on silage when mixed with legume *Stylosanthes guianensis* compared to silage prepared from Guinea grass only. Furthermore, the inclusion *Leucaena* of 30% to the base material of native grasses silage decreased the CF content of the silage [26]. Silage with low CF can increase their value by increasing degradation in the rumen and leading to more benefits for consumed cattle.

In addition, the increase in molasses as a silage additive significantly decreased the CF content of corn stover silage (*p* < 0.05). The decline trough by the high addition of molasses leads to a high growth rate of LAB; hence more CF could break down, which finally decreased the silage CF content. Putra et al. [28] stated that the decrease in the CF of silage is due to the hemicellulose hydrolysis process that takes place during the ensiling process. However, unfortunately, due to limitations in our research, there was no testing on the growth rate of LAB during the ensiling process.

The current result showed that the interaction of *Leucaena* proportion and a dose of molasses significantly affected (*p* < 0.05), but the dose of molasses partially was not significantly affecting the hemicellulose content of corn stover silage. The hemicellulose content values for each treatment are presented in Table 2. The hemicellulose content decreased with increased *Leucaena* proportion (*p* < 0.05). Compared to other treatment interactions, it was partly owing to a decrease in the contribution of hemicellulose due to the addition of *Leucaena* but also caused by the hydrolysis of hemicellulose during ensiling processes. The hydrolysis of hemicellulose during the ensiling process is intended to make it more soluble, which can then be used as needed to meet fermentation requirements. As Widiyastuti et al. [29], stated that three possibilities caused the degradation of hemicellulose, i.e., 1) degraded by the hemicellulose enzyme of the plant itself, 2) degraded by the hemicellulose enzyme bacteria, and 3) hydrolyzed by organic acids during fermentation processes.

The higher proportion of *Leucaena* in corn stover silage indicated lower ADF and NDF content. A similar effect also showed by molasses; the higher molasses added, the lower the ADF and NDF content of corn stover silage. Their combination also significantly affected NDF content (*p *< 0.05). Although, somehow, the combined effect has no significance on the NDF content of the corn stover silage. The ADF and NDF contents in this experiment are closely related to the decrease of CF of corn stover silage after ensilage processes.

The decreased content of ADF and NDF obtained in this study can be illustrated by the disruption of the complex lignin-carbohydrate during ensilage. Microbes degrade the released soluble carbohydrates to meet their needs, as explained in the CF section in this paper. Cellulose, hemicellulose, lignin, and silica are the constituent components of ADF. Dilaga et al. [23] explained that the low content of the ADF fraction obtained in their study was due to the ability of microbes to separate hemicellulose-lignin linking to making up the cell walls, and part of the hemicellulose was also degraded, causing the low content of the ADF fraction. However, the discussion regarding reducing the CF fraction in silage is still incomplete and needs further study.

### Silage fermentability quality

The result showed that the pH of corn stover silage was significantly affected by the addition of *Leucaena* proportion and the dose of molasses (*p* < 0.05), but there was no recorded interaction effect between treatment factors. The increase of *Leucaena* proportion has followed the increase in pH value (3.58 *vs.* 3.65 *vs.* 3.89 *vs.* 4.07; for each treatment, *p* < 0.05). This condition clearly showed the buffer capacity of protein components of silage. Other researchers also showed that there was an increase in the pH value in corn stover silage with a mixture of Common Veth and Alfafa legumes [30]. The high buffer capacity of particular feed material will require much more acid as an agent of conversion and vice versa. The critical pH for silage is about <4.5. The pH range produced in our study met these criteria. The low pH of silage can prevent undesired microorganisms from competing for the use of fermented sugar, pursuing other fermentation pathways, and producing a variety of metabolic products. The materials that have been ensiled have an almost neutral pH, and the substrate for fermentation is made of raw materials [24], as well as from outside inputs like silage additives. 

Due to its low price and constant availability, molasses was considered the most excellent sugar substrate for silage preservation. Adding molasses in silage affected the increase of glycolytic activities, to produce lactic acid as a fermentation product, LAB could use the hydrogen ions (H^+^) availability as an electron acceptor. The low pH value in silage indicated the dynamics of the fermentation during the ensiling process [31] one of which can determine the production of lactic acid and may have prevented protein degradation during the ensiling process. However, in our study, no interaction effect was observed between *Leucaena* and molasses on the pH value of corn stover silage.

The good indication of silage preservation during fermentation is indicated by ammonia nitrogen, a component of the non-protein in silage [32,33]. The concentration of NH_3_-N in the silage relates to protein degradation caused by plant enzymes or the activity of microbes, particularly microbial enzyme activities. The Ammonia nitrogen silage concentration in each treatment of L0, L15, L30, and L45 showed an increase in NH_3_-N concentration (5.53, 6.24, 6.90, and 8.27 mg/100 ml (*p* < 0.05). This result is similar to the effect reported by Bureenok et al. [27]. There was associated with the high buffer capacity of legumes that supported the production of other organic acids except for lactic acid [34]. The decrease in pH affects the formation of NH_3_-N because there was no hydrolysis of protein which means the lower NH_3_-N concentration in silage. Ammonium concentration of silage was significantly affected by molasses (*p* < 0.05); as described earlier, molasses added to silage possess a vital role as an energy source for epiphytic LAB, that form from growth modulation processes. Cazzato et al. [35] reported that the inoculation of *L. plantarum *in silage significantly suppressed the NH_3_-N formation. LAB, which was formed, provides advantages in decreasing pH, ammonium, and butyric acid production, and increasing lactic acid concentration [36,37].

### In vitro DMD and OMD 

Digestibility is the number of feed ingredients that can be digested by the digestive tract of livestock in the rumen and then absorbed by livestock in the small intestine. The results of our study showed that the DMD and OMD of silage increased linearly with the increasing portion of the addition of *Leucaena* (*p* < 0.05). This increased digestibility is owing to a decrease in CF due to an increase in the portion of *Leucaena*. The presence of legumes in the feed provides a source of nitrogen for rumen microbes. The available nitrogen source can promote cell multiplication for them with the availability of carbon and ATP. Kariyani et al. [7] described mixed *Leucaena* and cassava chips with a maximum level inclusion of 47.5% and cassava pulp with a maximum level inclusion of 28% achieved high live weight gain in Bali cattle. *Leucaena* base diet without mixed with maize stover produced a digestibility of 60.6% while combining *Leucaena* with maize stover had a digestibility of 58.8% with a mixture ratio of 75:25 [38]. In the context of silage, our research results were confirmed by [28,39] also reported that adding 20%–40% of legume in silage significantly increased the digestibility of silage.

Likewise, the effect of the dose of molasses treatment was an increase in the digestibility value of corn stover silage (*p* < 0.05). Thus, increasing portions of *Leucaena* and molasses in corn straw silage will impact the DMD and OMD of corn stover silage. The high DMD in the feed indicates the quality of this feed. The current results of our study also showed a significant interaction of both treatment factors on the increased digestibility of OMD and OMD of corn stover silage (*p* < 0.05). The increased digestibility of corn stover silage in our study was due to the availability of sufficient N sources of protein origin, which was positively correlated with a decrease in CF content as well as the availability of soluble carbohydrates, which supported a faster rumen microbial proliferation which in turn improved overall rumen performance. Qu et al. [40] suggest that higher protein content and lower fiber content in legumes than in grass may affect digestion.

Overall, the interaction effect between *Leucaena* and molasses addition used in this study needs to be looked at as directly as it might have a significant influence on animal nutrition. Therefore, predicting the subsequent association effect on cattle output is extremely challenging. As a result, the finding of this association effect provides an opportunity for future research to establish how these changes in mixed silage impact cattle production.

## Conclusion

This study shows that the inclusion of *Leucaena* in 30%–45% is very effective in increasing and improving the chemical composition of corn stover silage because this proportion significantly suppresses the content of CF and its fractions and increases the CP content of the silage. Likewise, the inclusion of molasses at a dose of 4% also positively contributed to the quality of the resulting silage, especially its effect in suppressing the buffer capacity of proteins resulting in low pH values and NH_3_-N concentrations in silage. Overall, there was a synergistic interaction between *Leucaena* and molasses in increasing the chemical composition, silage fermentation quality, and improving the rumen digestibility with the best combination obtained at the proportion of *Leucaena* of 45% with a dose of molasses of 4%. Further, an *in vivo* study should be carried out to investigate the direct effect of increasing the proportion of *Leucaena* and the dose of molasses in corn stover-based silage, especially the effect on overall production performance.

## List of abbreviations

L, *Leucaena*; M, Molasses; LAB, Lactic acid bacteria; DM, Dry matter; OM, Organic matter; CF, Crude fiber; CP, Crude protein; ADF, Acid detergent fiber; NDF, Neutral detergent fiber; WSC, Water-soluble carbohydrate; NH_3_-N, Ammonia; DMD, Dry matter digestibility; OMD, Organic matter digestibility.
